# Virtual Group Cognitive Stimulation Therapy for Dementia: Mixed-Methods Feasibility Randomized Controlled Trial

**DOI:** 10.1093/geront/gnae063

**Published:** 2024-06-06

**Authors:** Aimee Spector, Nur Diyanah Abdul Wahab, Joshua Stott, Emily Fisher, Esther K Hui, Luke Perkins, Wing Gi Leung, Rachel Evans, Gloria Wong, Cerne Felstead

**Affiliations:** Department of Clinical, Health, and Educational Psychology, University College London, London, UK; Department of Clinical, Health, and Educational Psychology, University College London, London, UK; Department of Clinical, Health, and Educational Psychology, University College London, London, UK; Department of Clinical, Health, and Educational Psychology, University College London, London, UK; Department of Clinical, Health, and Educational Psychology, University College London, London, UK; Department of Clinical, Health, and Educational Psychology, University College London, London, UK; Department of Clinical, Health, and Educational Psychology, University College London, London, UK; University of Bangor, North Wales Medical School, Bangor, UK; Department of Social work and Social Administration, University of Hong Kong, Kong Kong, Kong Kong; Department of Psychology, Leeds Teaching Hospitals NHS Trust, Leeds, UK

**Keywords:** Intervention, Psychosocial, Technology

## Abstract

**Background and Objectives:**

Cognitive stimulation therapy (CST) is an evidence-based group intervention for people with dementia, with benefits for cognition and quality of life when delivered face-to-face. Many people are unable to attend face-to-face groups for reasons including health and transport issues. This study aimed to assess the feasibility and acceptability of online or “virtual” CST (vCST).

**Research Design and Methods:**

Single-blind, randomized controlled feasibility design with qualitative interviews. Forty-six people with mild-to-moderate dementia were randomly allocated to attend either 14 sessions of twice-weekly vCST (*n* = 24) or treatment as usual (TAU, defined as usual care; *n* = 22) over 7 weeks. Cognition, quality of life, and depression were assessed pre- and posttreatment. Qualitative interviews (*n* = 16) with participants and carers were analyzed using thematic analysis.

**Results:**

High levels of attendance, adherence, fidelity to the manual, and completion of outcomes were recorded. Recruitment appeared feasible although randomization may not have been acceptable to some. There were no statistical differences noted between vCST and TAU in any of the outcomes evaluated, although both quantitative and qualitative data indicated acceptability, with qualitative reports of improved outcomes including cognition.

**Discussion and Implications:**

vCST appeared feasible to deliver but did not result in any changes in outcomes, as expected from an underpowered feasibility trial. CST is the main psychosocial intervention delivered for dementia in UK memory services and globally, with many services moving towards virtual CST delivery. Therefore, a fully powered RCT of the effectiveness of vCST is feasible and justified.

Dementia is a significant cause of disability and in the absence of a cure, there is heavy reliance on the provision of both pharmacological and psychosocial interventions. Cognitive stimulation therapy (CST) is a brief, clinical, and cost-effective group intervention for people with mild-to-moderate dementia (Spector et al., 2003) and the only nonpharmacological intervention recommended by the UK National Institute for Health and Care Excellence (NICE, 2018) guidelines to improve cognition, independence, and well-being. CST typically consists of 14, 45-min sessions over 7 weeks (Spector et al., 2003). Developed in the UK, it is now used worldwide (International CST Centre, 2023). CST aims to improve cognitive function through themed group activities, which implicitly stimulate skills including memory, executive function, and language through tasks such as categorization, word association, and discussion of current affairs. It is built upon several theories including learning theory and brain plasticity (Skaper et al., 2017), which suggest that appropriate and targeted mental stimulation, for example, through building new semantic connections, can lead to the development of new neuronal pathways. Social theories suggest that creating an optimal and supportive group environment can enhance skills and increase well-being (Kitwood, 1997). Global clinical trials confirm significant impact on cognition, quality of life (QoL), and mood and that group delivery is essential for significant benefits (Woods et al., 2023). Qualitative studies demonstrate social impact, for example, in confidence and developing relationships (Gibbor et al., 2020). Being a “type one standard” of the UK Memory Services National Accreditation Programme (MSNAP), all MSNAP-accredited NHS memory services (MSNAP, 2022) are now obliged to offer CST to people diagnosed with dementia. CST is typically the only nonpharmacological postdiagnostic treatment offered.

During the coronavirus disease 2019 (COVID-19) pandemic, access to face-to-face psychosocial interventions for people with dementia was restricted and many services rapidly shifted to delivery through digital technology, using platforms such as Zoom (Cuffaro et al., 2020). Whilst there are well-documented inequalities in access to digital technology for older people and those with dementia (Pywell et al., 2020), the move to digital delivery also highlighted the gap in service provision for people who were not able to access face-to-face services, including those with reduced mobility, who cannot readily access transport, and those living in rural communities (Cuffaro et al., 2020). In response to this situation, a multidisciplinary team of researchers codeveloped an international protocol for the virtual delivery of CST. The protocol was field-tested with 10 groups of people with dementia in Brazil, China (Hong Kong), India, Ireland, and the UK, with feedback gathered from 14 facilitators. Field testing in the five countries indicated acceptability to group facilitators and participants, with feedback used to refine the protocol (Perkins et al., 2022). Meanwhile, a team from New Zealand documented their success in moving CST groups online during the pandemic (Cheung & Peri, 2020). A survey of 33 UK memory services (Fisher et al., 2023) found that during the period of COVID-19 restrictions, 55% offered CST group sessions virtually through video conferencing platforms. Critically, 80% of services stated that they planned to continue with a hybrid model long-term, with the remaining 20% intending to deliver face-to-face CST only.

A recent meta-analysis which included 283 studies showed that Cognitive Stimulation was one of only three types of interventions associated with less frequent nursing home admission in older people (Gaugler et al., 2023). This, in addition to CST being the main nonpharmacological intervention delivered to people with dementia, make an understanding of its feasibility when delivered virtually (as well as potential benefits) critical. Using the protocol of virtual CST (vCST) described above, the current study employed both quantitative and qualitative methodology, aiming to assess:

1. Whether this vCST protocol is feasible and acceptable to deliver to people with dementia.
2. The potential impact of vCST on cognition, mood, and QoL.
3. The feasibility of conducting a randomized controlled trial (RCT) evaluating vCST to inform the design of a future definitive RCT.

## Method

### Overview

Following the Medical Research Council framework for the development of complex interventions (Skivington et al., 2021) and having completed stage 1 (development of an intervention) (Perkins et al., 2022); this current study reports on phase 2 (feasibility and piloting) using an RCT design with nested qualitative interviews. The trial was registered with ClinicalTrials.Gov, identifier: NCT04695743 and unique Protocol ID: 17127/002 and was guided by an established framework for conducting feasibility studies (Eldridge et al., 2016).

### Ethics and Sample Size

Ethical approval for the study was received from the University College London Research Ethics Committee (Project ID: 17127/001). All participants provided informed consent prior to participation. They were informed that they could withdraw at any point without having to give a reason and their capacity and consent to participation were reviewed throughout the study. Sample sizes between 24 and 50 have been recommended for feasibility studies (Julious, 2005; Sim & Lewis, 2012), although there is no consensus on an exact figure for feasibility trials (Billingham et al., 2013). Based on these recommendations, we aimed to recruit between 40 and 50 participants. A sample of 50 people would allow adequate precision around our expected retention rate (80%) to within an 85% confidence interval of ±11% (NIHR web link).

### Recruitment and Inclusion Criteria

People with dementia were recruited through third-sector organizations, such as the London Memory Services Network Group, Memory-Matters, Age UK, Camden Carers, and the Join Dementia Research network (JDR, an online recruitment platform). People who indicated an interest in participating, or whose interests and demographics matched our study inclusion criteria on the JDR network, were contacted via telephone or video conferencing for further screening of eligibility. Thereafter, participants and their carers provided informed consent if they were interested and met our eligibility criteria.

Inclusion criteria comprised:

- Diagnosis of dementia of any subtype, according to the International Classification of Diseases-10 (ICD-10; World Health Organization, 1993), of mild-to-moderate severity.
- Able to communicate verbally in English.
- Able to engage and participate in an online group for 1 hr.
- Capacity to provide informed consent to complete study measures and to video recording of sessions.
- Access to and ability to use a device capable of video conferencing.
- Access to an internet connection.

Exclusion criteria comprised:

- Accessing any other psychosocial intervention or psychological therapy for the duration of the trial.
- Having participated in a CST research program within the past 6 months.

### Procedure

**Figure 1.**
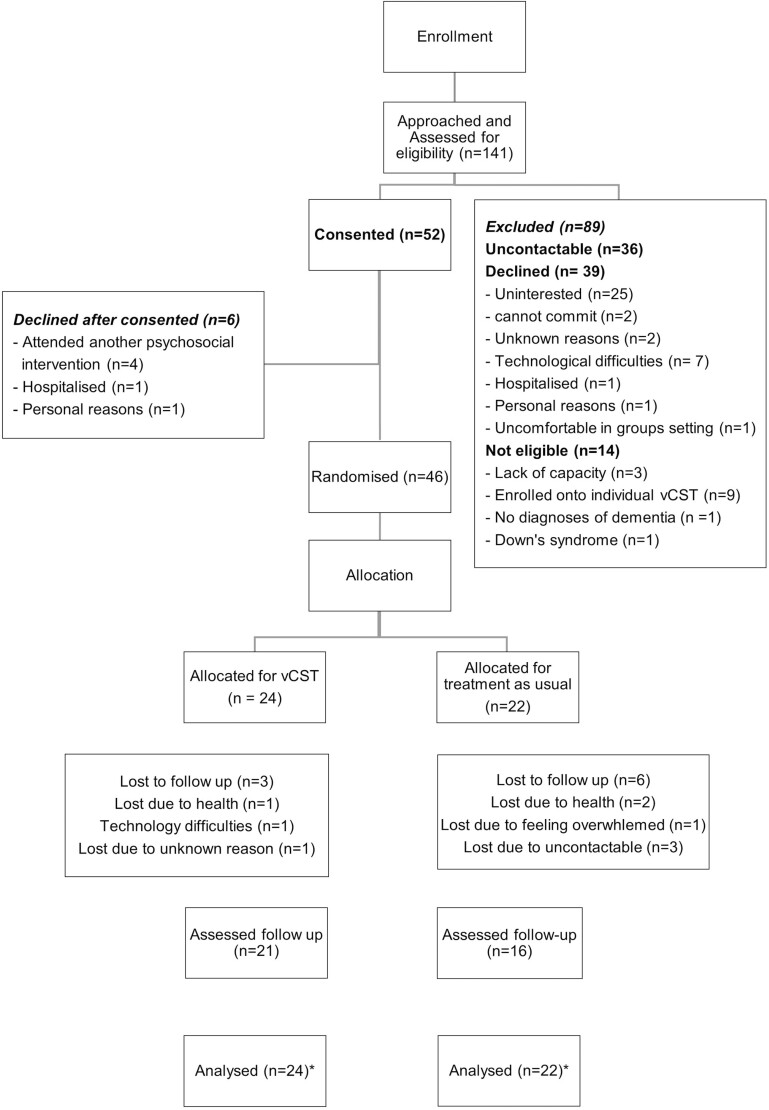
Recruitment and retention flow diagram. vCST = virtual Cognitive Stimulation Therapy. * Multiple imputation method was used to impute missing data for the secondary, explorative analysis.

Of the 141 people with dementia who were contacted, 105 people were screened for eligibility, and 46 were recruited into the trial. See Figure 1 for a description of the study flow, as guided by CONSORT (Schulz et al., 2010). The main reasons for not being recruited were being uncontactable (36/89) or not being interested when given further information (25/89). The process of gaining informed consent was guided by the Mental Capacity Act (2015). This included the use of information sheets and consent forms, within which participants were informed that they could withdraw from the study at any time with no consequences. Once a minimum number of eight people had consented to the trial, baseline assessments were conducted and all were randomized to either vCST or TAU. Randomization was conducted using a web-based randomization tool and Microsoft Excel to generate randomized codes in a 1:1 ratio by a researcher not directly involved with data collection or group facilitation. Convenience sampling was used, whereby all participants were invited to qualitative interviews. The total sample size of 11 people with dementia, with five accompanied by carers (hence total *n* = 16), was informed by concept of information power and pragmatics.

### Intervention

The vCST intervention involved 14, 60-min group sessions delivered twice a week for 7 weeks. Session materials were adapted from the existing face-to-face CST protocol (Spector et al., 2003) for online administration by Zoom, following the same 14-session themes (e.g., “physical games,” “being creative,” “childhood,” “food,” and “current affairs”). Groups consisted of four people with dementia and two facilitators, all assistant or trainee psychologists who had attended a CST training course and/or had delivered CST previously. “How-to” guides, which provided four steps to joining Zoom meetings (including images of the screen) were provided to participants unfamiliar with using Zoom. Further, the cofacilitator offered technical support during sessions if needed.

### Treatment As Usual

Treatment as usual (TAU) was defined as the care currently being received by participants in their day-to-day contexts. This was likely to include medication but due to COVID-19, all day services were suspended. Anyone receiving another psychosocial intervention for the duration of the trial was excluded.

### Feasibility and Acceptability

Feasibility of recruitment and retention was assessed by (i) successful recruitment of the target population within a period of 10 months; (ii) a retention rate of ≥75% from recruitment, through follow-up data collection in both groups; and (iii) full completion of ≥75% of the outcome measures. Acceptability of randomization was determined by the target randomization sample being reached. Acceptability of the intervention was assessed by (i) overall attendance and retention rates amongst the vCST participants (over 60%), (ii) any negative or adverse events related to the intervention, and (iii) qualitative feedback.

Fidelity was assessed by inviting all facilitators to complete the fidelity checklists following each session. As the fidelity checklist was developed alongside this trial, two versions were completed by three group facilitators, with version two being an updated version of version one. There were 15 items in version 1 and 16 items in version 2, with a total score of 0–33 and 0–34 respectively. The mean fidelity score and the percentage of the fidelity scores (fidelity score/total score of the checklist × 100%) were calculated for each vCST session. Based on criteria by An et al. (2020), 80% to 100% adherence to the fidelity checklist was interpreted as high fidelity, 51% to 79% as moderate and 50% or below as low fidelity (i.e., >80% of items on the checklist were implemented).

### Qualitative Interviews

The semistructured qualitative interviews were conducted via video call on “Zoom” with an independent researcher within a week of the final session to limit issues around recall. Some participants chose to attend the interview alone, while others attended jointly with their carer. Interviews lasted approximately 30 min with questions and prompts relating to three main areas: general experiences of the group; specific questions about CST as delivered online; and barriers and facilitators to accessing the intervention. An interview schedule was developed based on previous literature on qualitative experiences of CST (Orfanos et al., 2020), and in consultation with experienced members of the research team.

### Outcome Measures

Outcome measures were selected based on previous CST trials, with the key domains assessed (cognition, QoL, and mood) being those most sensitive to change in the CST literature (Lobbia et al., 2018). Pretest assessments were conducted before randomization and within the week before vCST groups, and posttest assessments were conducted within the week following the end of groups by researchers blind to group allocation. Standardized measures were administered in line with face-to-face administration through the use of the screen sharing function on “Zoom.”

#### Cognition

The Alzheimer’s Disease Assessment Scale-Cognitive Subscale (ADAS-Cog; Rosen et al., 1984) consists of 11 tasks assessing various cognitive domains, including memory, language, attention, command understanding, praxis, orientation, spoken language, and comprehension. A lower score indicates better cognitive performance and it has shown to have good reliability and validity (Sheehan, 2012). The Montreal Cognitive Assessment Blind (MOCA-BLIND) is similar to the original MOCA, only visual items are removed, enabling assessment over the telephone (Dupuis et al., 2014). It consists of six tasks, assessing cognitive domains including orientation, attention, memory, language, and abstract thinking, with a total score of 22, with higher scores indicating better cognitive performance. The MOCA-BLIND has been found to have good reliability and validity (Dawes et al., 2019).

#### Quality of life

The quality of life in Alzheimer’s disease (QoL-AD; Logsdon et al., 2002) is a 13-item measure assessing QoL in domains including physical health, energy, family, and living situation, with each rated on a 4-point Likert scale (“poor,” “fair,” “good,” and “excellent”). A higher score indicates a better QoL. The QoL-AD has been found to have good internal consistency, reliability, and validity (Thorgrimsen et al., 2003).

#### Depression

The Geriatric Depression Scale short form (GDS-15; Sheikh & Yesavage, 1986) is a brief screening tool consisting of 15 items to be rated as “yes” or “no,” with higher scores indicating greater depressive symptomatology. The GDS-15 has acceptable sensitivity, specificity, reliability, and validity (Herrmann et al., 1996; Marc et al., 2008; Weeks et al., 2003).

### Data Analysis

Primary analysis was conducted by assessing the feasibility data using the parameters outlined above through descriptive statistics.

Qualitative data were analyzed using a thematic analysis approach following the guidance outlined by Braun and Clarke (2019), a recursive process of extracting patterns across the data set. A bottom-up “inductive” approach to data analysis was adopted to identify themes. Themes were identified from transcripts at a “semantic” level, based on the surface-level meaning of participants’ spoken words.

Familiarization of the data was gained through the transcription process and by rereading all interviews. Initial ideas were transformed into “broad-brush” codes, organizing the data into meaningful groups. An iterative process of reviewing and refining the coded data into themes commenced until a thematic framework was finalized. Credibility checks were completed by an independent researcher on a random sample of the transcripts by reviewing and providing feedback on initial codes, with any discrepancies or disagreements resolved through discussion.

Exploratory analysis of quantitative outcome measures was conducted using the IBM Statistical Package for the Social Sciences version 28.0. Independent samples *t* tests and Chi-square tests examined any significant differences between the vCST and TAU groups for demographic variables and characteristics. A Fisher’s exact test was conducted for variables where 20% of cases had expected frequencies of less than five (Kim, 2017). An independent samples *t* test, or (where appropriate due to *t* test assumptions not being met) a Mann–Whitney *U* test was conducted to explore differences between groups for baseline outcome measures.

For the main quantative analysis, a mixed-model ANOVA using intention-to-treat was used to explore each outcome. Data were assessed for statistical assumptions of ANOVA including normality, homogeneity of variance, and sphericity. A Little’s test of Missing Completely at Random (MCAR) was not significant (χ^2^ 14.287, *df* = 21, *p* = .86), hence data were assumed to be MCAR. Adopting a multiple imputations methodology, the Markov Chain Monte Carlo method was used to impute missing values.

## Results

Forty-six participants were randomized of which 24 were allocated to vCST (six groups of four participants) and 22 to TAU. Of the 46 participants, 37 were assessed at follow-up, including 21 vCST and 16 TAU participants. Figure 1 shows the flow of participants through the trial and reasons for dropout. Overall, there was a 19.6% dropout rate.

Participant demographics are presented in Table 1. The age of the sample ranged from 48 to 88 years (mean 71.39), with equal numbers of males and females. All participants took part in the study online in their own homes using computers or electronic tablets that enabled them to join the session via Zoom. The analysis indicated that there were no significant differences between the vCST and TAU groups on demographic variables of age, education, gender, and ethnicity, as well as baseline QoL-AD and GDS-15 scores.

**Table 1.** Participant Demographics at Baseline

Characteristics	All participants (*n* = 46)	vCST (*n* = 24)	TAU (*n* = 22)
*Age (years)*			
Mean (*SD*)	71.39 (9.164)	71.96 (9.18)	70.77 (9.32)
Range	48–88	48–84	56–88
*Ethnicity (%)*			
German	1 (2.2)	1 (4.2)	1 (4.5)
Mixed White and Black Caribbean	1 (2.2)	0 (0)	1 (4.5)
White American	1 (2.2)	1(4.2)	0 (0)
White British	33 (71.7)	15 (62.5)	17 (77.3)
White European	1 (2.2)	1 (4.2)	0 (0)
White Irish	7 (15.2)	5 (20.8)	2 (9.1)
White Scottish	1 (2.2)	1 (4.2)	0 (0)
White (other)	1 (2.2)	0 (0)	1 (4.5)
*Gender (%)*			
Male	23 (50)	12 (50)	11 (50)
Female	23 (50)	12 (50)	11 (50)
*Years of education (%)*			
Less than 12 years	28 (60.9)	13 (54.2)	15 (68.2)
More than 12 years	18 (39.1)	11 (45.8)	7 (31.8)
*MOCA-BLIND Score*			
Mean (*SD*)	14.63 (4.36)	14.46 (4.90)	14.82 (3.79)
*ADAS-Cog Score*			
Mean (*SD*)	14.10 (9.98)	14.97 (10.43)	13.15 (9.62)
*QOL-AD Score*			
Mean (*SD*)	35.70 (6.68)	35.71 (6.87)	35.68 (6.62)
*GDS-15 Score*			
Mean (*SD*)	4.26 (3.71)	4.17 (3.41)	4.36 (4.09)

*Notes*: ADAS-Cog = Alzheimer’s Disease Assessment Scale-cognitive subscale; GDS-15 = Geriatric Depression Scale short form; MOCA-BLIND = The Montreal cognitive assessment—Blind; QoL-AD = Quality of Life in Alzheimer’s Disease questionnaire; *SD* = standard deviation; TAU = treatment as usual; vCST = virtual Cognitive Stimulation Therapy. *n* = number of participants.

### Feasibility of Recruitment and Retention

One hundred and forty-one participants were approached within a 10-month recruitment period, of which 52 agreed to participate and 46 were assessed and randomized. Thirty-nine declined the study invitation and 14 were not eligible for reasons including not having a diagnosis of dementia, having Down’s syndrome, unable to retain information to provide consent, and enrolled in another psychosocial intervention. Hence, the recruitment rate was 36.9% and eligibility rate was 99.01%. Overall, the study had a high retention rate of 80.4%, whereby 46 people with dementia completed baseline assessments and 37 completed the follow-up study (see Figure 1). Key reasons for losing people at follow-up were health, issues with technology, and being uncontactable.

### Acceptability of Randomization

The target randomization sample of 40–50 was reached, demonstrating that randomization was likely to be acceptable. Of note, there was a higher dropout rate in the TAU group (*n* = 6, 27.2%) compared to the vCST group (*n* = 3, 12.5%). Three of the six dropouts in the TAU group were uncontactable at follow-up assessment stage, making it impossible to ascertain whether the reasons were due to randomization or chance.

### Acceptability of the Intervention

Of those who did not drop out of the study, attendance rates were as follows: 11 completed all 14 sessions, seven attended 13 sessions, and three attended 12 sessions of vCST. Hence, mean attendance rate was high, with an average of 11.54 sessions (*SD* = 0.50; median = 14). Reasons for missing sessions were medical appointments, personal commitments, caregivers not available on the day and forgetting the session. Three participants dropped out of the study after two sessions. One reported struggling with technical problems, one’s dementia progressed and one lost interest in the study. One participant from TAU had a fall and was unable to complete a follow-up assessment. No adverse events were reported by any participants.

### Fidelity

The total average fidelity score of version one checklist was 27.1 (73.2%, *SD* = 5.61; range = 0–37) and 33 for version 2 (86.8%, *SD* = 3.76; range = 0–38). The average percentage was 80%, meaning that an average of 80% of the items on the fidelity checklist were implemented during vCST. This indicated high fidelity.

### Acceptability of Outcome Measures

Excluding the participants who did not complete the follow-up test, all participants completed all questionnaires at both baseline and follow-up (100% completion and no missing data), indicating that measures were acceptable.

### Qualitative Results

Sixteen people took part in qualitative interviews, 11 people with dementia and 5 carers. The mean age of the people with dementia was 73, with four males and seven females. Six participants attended the interviews independently, whilst five chose to attend along with their carer who contributed to the feedback (see Table 2 for a summary of the sample). Of the carers, there were two husbands, two wives, and one daughter. The thematic analysis generated five over-arching themes: “Acceptability of technology”; “CST processes in a virtual modality”; “Perceived positive outcomes”; “Connections with others”; and “Feelings about vCST,” with 12 subthemes (see Table 3).

**Table 2.** Qualitative Interviewee Demographics

Participant	Cohort	Attendance	People with dementia Demographics—Gender, Age, Dx.	At interview
1/2	1	100%	Female, 80, AD	People with dementia and carer (husband)
3	1	93%	Female, 66, AD	People with dementia only
4	1	100%	Male, 71, AD	People with dementia only
5/6	1	86%	Male, 71, AD	People with dementia and carer (wife)
7	2	93%	Female, 79, Mixed (AD and VD)	People with dementia only
8	2	100%	Female, 80, Mixed (AD and VD)	People with dementia only
9	2	93%	Female, 60, Mixed (AD and PCA)	People with dementia only
10/11	2	100%	Female, 66, AD	People with dementia and carer (husband)
12/13	3	86%	Male, 67, AD	People with dementia & carer (wife)
14/15	3	100%	Female, 84, AD	People with dementia and carer (daughter)
16	3	100%	Male, 77, unspecified	People with dementia only
Average		96%		

*Notes:* AD = Alzheimer’s disease; Dx = diagnosis; PCA = posterior cortical atrophy; People with dementia = person with dementia; VD = vascular dementia.

**Table 3.** Thematic Framework and Prevalence

Main theme	Subtheme	Prevalence
1. Acceptability of technology	Positives about the technology	Very prevalent
	Benefits of online compared to face-to-face	Majority
	Negatives about the technology	Majority
2. CST processes in a virtual modality	Group set-up Group content	Majority Majority
3. Perceived positive outcomes	Improving well-being Providing routine Changes in cognition	Majority Minority Minority
4. Connections with others	Positives Negatives	Very prevalent Majority
5. Feelings about vCST	Positives	Very prevalent
	Negatives	Very prevalent

*Notes*: Majority = theme applies to more than half of the interviews (6–9); Minority = theme applies at up to half of interviews (3–5); Rare = theme discussed at only one or two interviews (1–2); Very prevalent = theme was discussed at all, or all but one of interviews (10–11).

#### Theme 1. Acceptability of technology

##### Positives about the technology

Experiencing minimal or no technical issues formed a prevalent subtheme and when issues were reported, they were mostly “minor” occurrences with quick resolution. A minority of both people with dementia and carers commented on how quickly they were able to learn new skills on Zoom. They also commented on the Zoom functions working well, including showing images/videos on shared-screen and using the whiteboard and PowerPoint resources.

##### Benefits of online compared to face-to-face

Benefits of not having to travel and meeting people from a wide geographical area were noted. Some enjoyed the comforts of home being close to hand, for example, not having to “dress up” or ease of access when feeling unwell.

If I had have been having to travel 10 or 15 miles somewhere and I was feeling under the weather, you know I might have “ummed and ahhed” and not gone. But because it was online and I was warm and comfortable at home, even though I didn’t feel particularly wonderful, I could still attend. So, in that respect… it’s great. [P]

##### Negatives about the technology

A majority mentioned that attendance was reliant on having a strong internet connection and on others to support them, at least in the initial stages. Some, including those with communication difficulties, commented on how it was generally more difficult to speak online compared to face-to-face, and on the time-lag that is experienced with video conferencing applications.

I was talking and then you came along, and we both ended speaking at the same time. If you were here in my house that would not happen. So, it makes it more strange and difficult. [P]

When you’re in a real-life situation you can see, you can read people and you know by their movements and their expressions when they want to speak. And that’s quite difficult to do … online. [P]

#### Theme 2. CST processes in a virtual modality

##### Group set-up

Some reported finding regular email reminders helpful, as well as one-off contacts with the facilitator if requiring support. Others found it enjoyable and acceptable bringing preprepared stimuli to the groups (e.g., photographs). A minority commented on group size, mostly finding the “smaller” size ideal for vCST.

It was lovely that it was a small group … and it was, very inclusive … they got to know each other as time went on, which I kind of think also helped as well. [C]

##### Group content

Only a minority commented on the individual activities or content of the group sessions, such as enjoyment of singing, many reporting that it was difficult to remember specifics. One person found that activities, which involved turn-taking worked best and another remarked that they would have appreciated more time dedicated to discussing living with dementia.

#### Theme 3. Positive perceived outcomes

##### Improved well-being

A majority spoke about positive change they noticed in themselves or the person with dementia, the most prevalent relating to overall well-being or mood.

I think she used to come out in good form after it. You know… the way when you’ve been somewhere and you’ve enjoyed yourself, even out for a good movie or something like that, like you feel uplifted slightly. [C]

It has been an absolute life-enhancing experience for me, the whole thing. [P]

After that … if he was in low mood in the morning, after that session he’d be happy. [C]

##### Providing routine

A minority commented on how groups had provided much needed structure or routine to their week.

It was something for him to focus on you know … And he’d be up and ready washed and dressed and just sitting there waiting for them to join. [C]

##### Changes in cognition

A minority noticed changes in cognition, including increased engagement with others, for example, talking about topics from the group, and also more coherent expressive language skills.

After some sessions [participant] was more, like lively, motivated and energised. [C]

He was able to make sense in the conversation when he came off it. [C]

#### Theme 4. Connections with others

##### Positives

Many expressed how they were able to connect with others in some way online and offered an antidote to isolation during COVID-19:

I felt a breadth of humanity, warmth from the facilitators and from the other participants, and oh my goodness when you feel as isolated as I do right now, that warmth and compassion can’t be measured, it’s just immeasurable, it was first class. [P]

A majority of those interviewed highlighted a bond they had formed with someone in the group and that the compatibility of group and group dynamics was affected by factors such as age and cognitive ability.

##### Negatives

One participant noted that they were not able to form relationships with others and a minority commented on missing the “intimacy” of face-to-face interaction including tactile aspects, being “physically” present and the unstructured social interaction time.

… Once the group’s finished you’re sat here by yourself. Whereas if you’re out with the group … you might walk to the bus-stop together or you may have arrived together or leave together, and sort of talk through what you have done that morning. Whereas when you’re online there is no one else really you can talk to about it, because there is just you. [P]

#### Theme 5. Feelings about vCST

##### Positives

“Enjoyment” was mentioned in all 11 interviews. Other common emotions related to a sense of “inclusion” and “interest.” A minority expressed feeling comfortable, safe, respected, looking forward to the groups, happiness, novelty, and a boost in confidence. Every participant stated that they would be willing to participate in online vCST again.

One carer expressed positive benefits to their own well-being, via “respite” time from caring, and in learning new skills to use technology:

Then my brain was able to switch off while I was sitting in the other room (laughs), so both of us gained… even sitting on the phone for an hour, I’d often ring my friends and I’d sit and be able to have a conversation without being interrupted. You know it worked out well. [C]

The majority of participants reported finding the group sessions mentally stimulating, whilst only one participant reported otherwise.

##### Negatives

A minority expressed missing the group once it was over and missing the journey to groups.

It’s the journey there and back… I don’t go to work anymore because of dementia, so it’s something to do isn’t it. So rather than having one hour just on screen, it would be a total of three hours taken care of. [P]

Two carers reported initial reservations about accepting something online but felt that it had in fact exceeded expectations.

At the beginning when they were saying do it online, I was nearly objecting to be honest with you, because lazy-brained I wouldn’t be that well up with the computer, but then when I started to get going, I was so happy that I chose to do it. [C]

It surprised me, I didn’t think it would be as successful as it was. [C]

Two participants felt that groups highlighted their cognitive difficulties when they were unable to do a task. A majority were clear on their preference for face-to-face groups over online, if provided with the choice.

Well, I definitely prefer face-to-face if that were a choice. But due to COVID, or generally speaking for some people who might be unable to leave their home or can’t access group-settings … I think it’s a perfect solution. [P]

### Quantitative Outcome Measures

For all outcomes, statistical significance was not achieved, as expected with such a small sample. Mean differences at follow-up have been evaluated based on *t* tests between treatment groups, these did not account for baseline scores.

#### Cognition

For the MOCA-BLIND a difference between groups of −0.096 was found, with the posttest mean difference between groups in favor of the vCST group.

For the ADAS-Cog, a difference between groups of −0.639 was found, with the posttest mean differences between groups in favor of TAU.

#### Quality of life

For the QoL-AD a difference between groups of 0.23 was found, with the posttest mean difference between groups in favor of TAU.

#### Mood

For the GDS, a difference between groups of −0.59 was found, with the posttest mean difference between group in favor of TAU.

### Power Calculations for Future Trial

Effect sizes, based on calculations provided in Table 4, were calculated to inform power calculations for a future trial. An estimate of the population standard deviation was obtained by pooling data from both the treatment and control groups. The effect size estimates that were calculated were corrected for bias (Morris, 2008). The estimates suggest small effect sizes for QoL-AD (*d* = 0.18) and GDS-15 (*d* = 0.061), using Cohen’s criteria (1988). G*power version 3.1 (Faul et al., 2007) was used to estimate a sample size for a future trial based on these effect sizes. To have 80% power to detect a significant interaction effect for a within-subjects ANOVA design (α = 0.05), a sample size of *n* = 246 is required for QoL and *n* = 2,112 is required for depression. It is, however, important to consider that the results of the current study are based on a small feasibility sample. It may therefore be more appropriate to power future vCST trials based on other trials of face-to-face CST. These effect sizes calculated should be used to inform the power calculation focusing on confidence intervals whilst incorporating minimal clinically important differences of the measures.

**Table 4.** Changes in Outcomes Between Groups

Variable	Pretest	Posttest	Pre-post-test mean difference between group (vCST vs TAU)	2 × 2 ANOVA	Effect size
					(Cohen's *d*)
MOCA-BLIND score (Mean [*SD*])					
vCST (*n* = 24)	14.46 (4.90)	14.33 (4.58)			
TAU (*n* = 22)	14.82 (3.79)	14.23 (3.67)	*t*(44) = −0.096, p = .92	*F*(1,44) = 0.011, *p* = .918	*d* = 0.02
ADAS-Cog score (Mean [*SD*])					
vCST (*n* = 24)	14.97 (10.42)	14.51 (11.43)			
TAU (*n* = 22)	13.15 (9.62)	12.54 (9.35)	*t*(44) = −0.639, p = .53	*F*(1,44) = 0.30, *p* = .587	*d* = 0.19
QoL-AD score (Mean [*SD*])					
vCST (*n* = 24)	35.71 (6.87)	37.30 (5.46)	*t*(44) = 0.23, *p* = .82		
TAU (*n* = 22)	35.68 (6.62)	37.70 (6.10)		*F*(1, 44) = 0.16, *p *= .70	*d* = 0.07
GDS-15 score (Mean [*SD*])					
vCST (*n* = 24)	4.17 (3.41)	3.52 (2.70)	*t*(44) = −0.59, *p* = .56		
TAU (*n* = 22)	4.36 (4.09)	3.02 (3.12)		*F*(1, 44) = 0.35, *p *= .56	*d* = 0.17
GDS-15 score; transformed (Mean [*SD*])					
vCST (*n* = 24)					
TAU (*n* = 22)	0.62 (0.31)	0.57 (0.29)	*U *= 229.50*, p *= .45		
	0.59 (0.39)	0.50 (0.32)			

*Notes*: ADAS-Cog = Alzheimer’s Disease Assessment Scale-cognitive subscale; *d = *Cohen’s *d;* GDS-15 = Geriatric Depression Scale short form; MOCA-BLIND = The Montreal cognitive assessment—Blind; *n* = number of participants; QoL-AD = Quality of Life in Alzheimer’s Disease questionnaire; *SD* = standard deviation; TAU = treatment as usual; vCST = virtual Cognitive Stimulation Therapy.

## Discussion

Results suggested that evaluating vCST within an RCT is feasible, and that vCST is an acceptable intervention for people with dementia. The research team successfully recruited the target sample within 10 months, although a few expressed feeling uncomfortable with technology or preferring an individualized intervention. Whilst there was higher dropout in TAU, it was not possible to ascertain whether this was due to chance or lack of acceptability of randomization. A retention rate of 80.4% showed that most people stayed in the study for the full duration. Session attendance rates were high overall, with an average of 11.54 sessions. 52.4% of participants received the full 14 sessions, and 87.6% of them completed at least 10 sessions. Fidelity was high (80%), and all outcome measures used appeared to be acceptable based on completion rates.

Qualitative results indicated that the online format was acceptable to most people. Regarding “Acceptability of technology,” “Feelings about vCST,” and “Connections with others” there were more positive reflections than negative. All of those interviewed, including those with more critical reflections, reported that they would join vCST groups again, indicative of a high degree of acceptability.

Despite this, the majority of participants on weighing up their preferences, reported that they would ultimately prefer face-to-face groups if given the choice. Reasons for this varied, yet many placed higher priority on meeting people “in real-life” than the convenience of attending online. Many remarked on the potential scope and benefits of vCST for “others,” including people with mobility issues, those unable to drive, or those with worsening physical illness.

As stated earlier, there is consistent evidence across multiple trials for the benefits of face-to-face CST across several outcomes including cognition, QoL, and depression (Woods et al., 2023). This current study did not find any changes and whilst this is expected in an underpowered feasibility trial, of note there were no positive trends in any outcome in favor of vCST. That said, the qualitative interviews revealed improvements observed by both people with dementia and carers in cognition, mood, well-being, confidence, language skills, and more active engagement with others. Relationship-building appeared possible within vCST, with the majority of participants describing developing friendships, closeness, and connection with others. It appears that the small group size of four participants and two facilitators helped to foster safety, and enabled participants to develop interpersonal bonds.

### Interpretation of Findings

Whilst a full RCT is required to establish effectiveness, as follows are some possible limitations of vCST compared to face-to-face delivery. vCST is dependent on a two-dimensional screen, limiting richness in multisensory cues and physical reciprocation. Attempts were made to compensate, for example, by asking people to bring physical objects to sessions and increasing the virtual social interaction before or after sessions. Nonetheless, the program was heavily focused on auditory and visual stimulation, with diminished stimulation of tactile, olfactory, and gustatory senses. Another explanation might be a ceiling effect, with baseline cognition in this study being higher than in other CST trials and limiting scope for improvement. The baseline MOCA score here was 14.63 (equating to an approximate MMSE score of 20; Fasnacht et al., 2023). This compares to a mean MMSE score of 14.4 in the original Spector et al. (2003) trial. The lack of significant QoL and mood effects may be associated with this lack of cognitive improvement, with previous research finding that the impact of CST on QoL may be mediated by cognitive improvements (Woods et al., 2006). In terms of mood, the one study that found the most notable impact (Marinho et al., 2021) included a fairly depressed sample at baseline, in contrast to this study which had low levels of baseline depression, potentially limiting scope for improvement.

In the initial development of vCST, it was found that people with dementia can find it more difficult or tiring to engage online compared to face-to-face, and that digital literacy affected engagement (Perkins et al., 2022). It is possible that participants had more difficulty recognizing or remembering each other on screen, or forming relationships, due to the lack of physical contact. Meeting the need for social engagement can improve symptoms of distress in dementia (Cohen-Mansfield, 2018), suggesting that engagement is a relevant factor to consider for improving outcomes.

The mean attendance rate was 11.54. This compares to 11.6 in the main face-to-face CST trial (Spector et al., 2003). Eighty-eight percent of the sample here completed seven or more sessions, compared to 89% in the face-to-face trial. This indicates that attendance to virtual CST groups might be similar in practice as to face-to-face groups, with a level of dropout always expected in a dementia sample due to reasons including comorbid physical health problems, availability of caregivers, and mortality.

### Limitations

Unblinding was not formally recorded; hence, there is no documentation of any accidental unblinding where participants inadvertently revealed their group allocation to assessors. However, given that there were no changes in outcomes between groups, this is unlikely to have been problematic. The majority of participants were of White ethnic background, and those lacking access to technology were excluded. It is likely that the sample was biased toward a more educated, higher socioeconomic status and will not be representative of the broader UK population. It is also possible that those who agreed to complete qualitative interviews were biased toward people who were more positive about the vCST experience.

There were limitations with the measures used, for example, the QoL-AD is not validated for online administration, and studies have found that the GDS-15 diminished in validity when administered to people with dementia compared to those without dementia (Kørner et al., 2006). Two versions of the fidelity checklist were implemented as it was still under development during the initial stage of vCST. Further, any self-report measure is subject to bias (the “Hawthorne effect”). We did not collect data on what “treatment as usual” (TAU) consisted of in both groups, which would have been helpful contextual information. The use of TAU as the control condition did not allow us to ascertain whether any change in outcomes was associated with the intervention or with support from the clinician and other nonspecific factors. Not considering mechanisms of action or fidelity to the treatment manual were further limitations.

For the qualitative interviews, there was reliance on people to provide detailed feedback on their experiences in the group, which was limited due to their memory problems. The omission of qualitative interviews from those who did not choose to participate in the study is a further limitation, particularly in relation to understanding barriers to participation.

### Implications for Future Research

Several future directions are suggested in light of the limitations above. Qualitative feedback indicated that sessions were enjoyable, stimulating, and convenient to attend at home (Perkins et al., 2022), and vCST appears feasible and acceptable. Therefore, a larger-scale, fully powered RCT is warranted and required to establish the effectiveness of vCST and to compare this with groups delivered face-to-face. The effect sizes found in this study may be used to inform the sample size for a future trial, along with studies based on other trials of face-to-face CST and other literature and guidance. A future trial should collect data on medication use, which may be considered in the analysis. Fidelity should be evaluated through independent observer ratings, in addition to self-reported fidelity, and a larger sample would enable exploration of potential mechanisms of action. Most fully powered RCTs would assume at least a 20% drop out rate; however, larger attrition rates can be incorporated (25%, 30%), which would increase the sample size. This should be discussed and considered during the development and design stage of the RCT. Whilst we experienced a 20% attrition rate, it would be useful to review other trials in this area to ensure the power calculation incorporates the appropriate level. Finally, a future trial should include an internal pilot study (including clear stop/review/go criteria) and embed a process evaluation to preliminarily examine mechanisms of change and identify areas for change if the trial fails to show efficacy.

Future studies on vCST could explore other outcomes perhaps more relevant to the target population, such as loneliness, anxiety, social engagement, or impact on caregiver; as this may provide a more accurate or holistic picture of benefits. Loneliness may be especially relevant given that virtual CST delivery aims to increase the accessibility of CST for people with difficulty accessing face-to-face interventions, and who may feel more isolated (Wickens et al., 2021). Lastly, it would be beneficial to consider the accessibility of vCST to a more representative population, including people from minority ethnic groups or of lower socioeconomic status; and to a more targeted populations, such as those from rural areas or with limited mobility.

### Implications for Practice

Cognitive stimulation therapy is becoming more widely implemented virtually in services in the UK; a recent survey of 33 memory clinics found that 80% of these clinics intended to offer a mix of virtual and face-to-face CST groups as a long-term option (Fisher et al., 2023). In the current study, several people with dementia and caregivers declined to join the study due to worries about setting up Zoom, having unstable internet, and lack of devices. Some group members were highly dependent on caregivers’ support and occasionally missed sessions due to caregivers’ unavailability. These factors would need to be considered when planning the wider implementation of vCST. There remains the question of whether the accessibility of vCST could be extended to people who do not have access to or knowledge of the appropriate technology, for example, through borrowing devices and teaching them to use them.

## Conclusion

The 14-session vCST for people with dementia was successfully implemented with high attendance and retention rate. A future RCT to demonstrate effectiveness appears feasible and warranted. vCST may offer an alternative CST treatment to people who have mobility or health difficulties and are geographically isolated, with qualitative outcomes indicating potential benefits to cognition and general well-being.

## Data Availability

Data are available upon reasonable request. The trial was registered with ClinicalTrials.Gov, identifier: NCT04695743 and unique Protocol ID: 17127/002.
